# Integrative Proteomic and Phosphoproteomic Analysis Reveals Altered Vesicle Transport in Systemic Lupus Erythematosus

**DOI:** 10.1186/s12014-026-09599-z

**Published:** 2026-04-28

**Authors:** Lingling Zhou, Yixi Li, Mengyao Wu, Donge Tang, Wei Zhang, Yong Dai

**Affiliations:** 1 School of Medicine, Anhui University of Science & Technology, Huainan, China; 2 Center for General Practice Medicine, Department of Rheumatology and Immunology, Zhejiang Provincial People’s Hospital (Affiliated People’ s Hospital, Hangzhou Medical College, Hangzhou, China; 3 Key Laboratory of Industrial Dust Deep Reduction and Occupational Health and Safety of Anhui Higher Education Institutes, Huainan, China; 4 Shenzhen People’s Hospital, The Second Clinical Medical College of Jinan University, Shenzhen, China; 5 Department of Clinical Laboratory, Peking University Shenzhen Hospital, Shenzhen, China

**Keywords:** Systemic lupus erythematosus, Proteome, Phosphoproteome

## Abstract

**Background:**

Vesicle transport genes (VTGs) are involved in the pathogenesis and progression of systemic lupus erythematosus (SLE). A comprehensive multi-omics analysis is crucial to elucidate their molecular alterations and identify potential biomarkers and therapeutic targets. However, studies investigating global alterations of VTGs in SLE remain limited. In this study, we aimed to investigate the relationship between VTGs alterations and SLE progression.

**Methods:**

We integrated proteomic and phosphoproteomic data from 130 SLE patients and 90 healthy controls (HC). This was combined with transcriptomic profiles from 1,461 SLE cases and 198 HC. Focusing on VTGs, our multi-omics analysis identified key phosphorylation events, stage-specific kinases, and transcription factor-target interactions. We then constructed signaling pathway networks for both the stable and active phases of SLE.

**Results:**

Proteomic analysis revealed altered expression across vesicle subclasses and marked dysregulation of critical processes such as organelle transport and autophagy in SLE. Phosphoproteomic profiling identified multiple aberrant phosphorylation sites and highlighted ITSN2 S889 as a potential hub phosphorylation site. Integrated analysis defined Clusters 4, 6, and 9 as early-altered molecules, and Clusters 1, 3, and 8 as progression-altered molecules. It also identified CLTC and its phosphorylated form T105 as candidate hub molecules. Multi-omics integration confirmed significant upregulation of HP and SAMD9 at both mRNA and protein levels, and implicated STAT1 and RELA as potential regulatory transcription factors (TFs). Based on their functional roles, kinases such as PKACB, SYK, PDGFRA, LCK, PKCA, PKCD, TBK1, AKT1, and DLK were implicated in the underlying pathogenic mechanism, whereas TAK1, AKT2, AKT3, and PITSLRE were associated with disease progression. A comprehensive signaling map capturing stage-dependent network alterations in SLE was constructed.

**Conclusions:**

This study contributes to a thorough understanding of the connection between alterations in VTGs and the development of SLE. It provides an integrated molecular map of VTGs dysregulation in SLE, suggesting potential opportunities for the development of diagnostic biomarkers and therapeutic interventions.

**Graphical Abstract:**

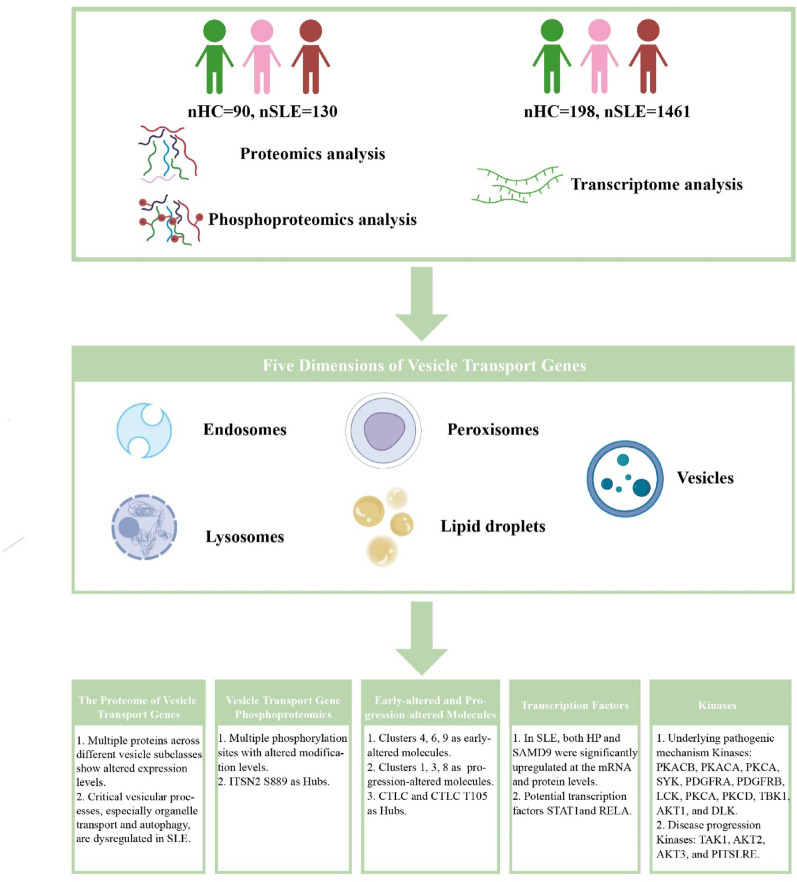

**Supplementary Information:**

The online version contains supplementary material available at 10.1186/s12014-026-09599-z.

## Background

Systemic lupus erythematosus (SLE) is a systemic autoimmune disease that predominantly affects women aged 20 to 40 years and is clinically characterized by multi-organ involvement. The pathogenesis of SLE arises from complex interactions among genetic, epigenetic, and environmental factors [1–3]. These factors collectively disrupt homeostatic regulation in immune cells and interfere with intercellular communication networks. In this pathological process, an imbalance in intracellular homeostasis directly impairs immune cell function, while dysregulated intercellular signaling promotes the activation and proliferation of autoreactive immune cells [4, 5]. These alterations ultimately lead to the production of pathogenic autoantibodies and immune complexes, causing multi-organ damage. Despite extensive research over decades, the precise regulatory mechanisms underlying cellular homeostasis and intercellular communication in SLE remain inadequately understood, highlighting the need for a systematic reevaluation of its pathophysiology from this perspective [6, 7].

In this context, cellular vesicles, which are key mediators of cellular homeostasis and intercellular communication, have drawn increasing attention due to their functional abnormalities and involvement in SLE pathogenesis [8, 9]. These lipid bilayer-based membrane structures contribute not only to maintaining intracellular homeostasis but also play an essential role as communication channels between cells. Research has shown that impaired vesicle function in SLE promotes disease progression by disrupting several critical biological processes, including antigen presentation, immune receptor internalization, signal transduction, and cytokine secretion [10–12]. Importantly, dysfunctions in specific vesicle subtypes, such as endosomes [13], lysosomes [14, 15], and peroxisomes [16], directly disturb the immune-metabolic balance, thereby aggravating autoimmune responses. These findings confirm that dysregulation of genes governing vesicular transport (VTGs) plays a central role in the pathogenesis of SLE and warrants further investigation.

Building on our previous exploratory profiling, which established a global proteomic and phosphoproteomic atlas of SLE PBMCs, the current study advances from broad-scale landscape mapping toward a hypothesis-driven interrogation of a specific functional axis. To systematically dissect the molecular mechanisms involving vesicles, we performed an integrated multi-omics analysis. Specifically, we analyzed proteomic and phosphoproteomic profiles of peripheral blood mononuclear cells (PBMCs) from 130 SLE patients and 90 healthy controls (HC), and complemented these data with RNA-Seq datasets from 1,461 samples obtained from the Gene Expression Omnibus (GEO). In contrast to our prior descriptive overview, the present study isolates vesicle transport as a coherent biological module. By systematically comparing vesicle transport-related genes (VTGs) across transcriptomic, proteomic, and phosphoproteomic levels, we identified key molecules closely associated with SLE progression and further classified them into early-altered and progression-altered categories. Finally, we reconstructed hierarchical regulatory networks linking upstream kinases and transcription factors to downstream vesicular effectors. This targeted approach enables a more granular understanding of how specific regulatory nodes—rather than broad pathway alterations—drive the loss of cellular homeostasis in SLE.

## Materials and methods

### Study cohort and PBMCs sample collection

This study utilized three independent clinical PBMC cohorts for proteomic and phosphoproteomic profiling, including 130 SLE patients categorized into stable (SLE_S, *n* = 82) and active disease (SLE_A, *n* = 48), alongside 90 matched HC. PBMCs samples were pooled into 36 protein pools (SLE_S: 14 pools, SLE_A: 7 pools, HC: 15 pools), with each pool containing 1 mg of protein (approximately 6 individual samples combined) for phosphoproteomic analysis. This pooling strategy was necessitated by the stringent protein input requirements for deep 4D label-free phosphoproteomics and the clinical constraints associated with lymphopenia in active SLE patients. Although pooling limits the assessment of inter-individual heterogeneity, it ensures the reliable detection of low-abundance phosphopeptides and facilitates the identification of robust, shared phosphoregulatory signatures at the population level by minimizing biological noise. All participants provided informed consent, in strict accordance with the Declaration of Helsinki. All SLE patients met the 2019 EULAR/ACR classification criteria, with disease activity assessed using the SLEDAI-2 K scale (SLE_S: SLEDAI ≤ 4; SLE_A: SLEDAI > 4). Patients with comorbid autoimmune diseases, infections, or malignancies were excluded. Healthy control samples were age- and sex-matched, with no history of immune-related diseases.

### Data acquisition and deposition

The proteomic and phosphoproteomic data used in this study were generated in our laboratory [3]. The datasets generated during and/or analysed during the current study are available in the ProteomeXchange repository with the accession number PXD025559. All differentially expressed phosphosites and proteins are listed in Supplementary Table S1-2. The mRNA expression matrices for PBMCs from patients with SLE were downloaded from the GEO. The datasets included GSE65391, GSE121239, GSE61635, GSE4588, GSE50772, GSE99967, and GSE24706. Following normalization, the datasets were consolidated, resulting in a combined total of 1461 SLE samples and 198 normal samples [17] (supplementary table S3).

### VTGs set

The VTGs set was obtained from the Human Protein Atlas. Gene annotations related to vesicle functions were searched and downloaded to ensure comprehensive coverage of genes associated with vesicle formation, transport, and functionality. The downloaded gene list was subsequently filtered and utilized for further bioinformatics analysis in this study (supplementary table S4).

### Protein-protein interaction (PPI) and subnet module

PPI network analysis was performed using the Metascape online platform (https://metascape.org) [18]. The list of selected differentially expressed proteins was submitted for analysis using the “Express Analysis” pipeline. Upon processing completion, the resulting analysis report was retrieved directly from the platform. To delineate functionally coherent modules within the interaction network, the MCODE algorithm was employed to identify densely interconnected protein clusters. Each identified cluster was subsequently annotated for its relevant biological functions.

### Hub proteins

The PPI network was first constructed using the STRING database [19]. Subsequently, core hub proteins were identified from this network by applying the Maximal Clique Centrality (MCC) algorithm via the cytoHubba plugin in Cytoscape software [20]. These proteins may play pivotal roles in cellular signal transduction and the regulation of biological processes.

### Mfuzz analysis

To identify the dynamic expression patterns of vesicle transport proteins and phosphoproteins in SLE patients and HC, time-series clustering analysis was performed on the proteomics and phosphoproteomics data from both groups. This analysis was conducted using the Mfuzz R package (version 2.64.0), which implements a fuzzy c-means algorithm to group proteins or phosphosites with similar expression dynamics into clusters for characterizing their temporal expression profiles [21].

### Upstream transcription factor prediction

We first selected VTGs that showed concordant expression at the mRNA and protein levels. Using the hTFtarget database (https://guolab.wchscu.cn/hTFtarget/#!/) [22], we subsequently identified tissue-specific TFs active in blood. Those TFs with changes in protein abundance or phosphorylation status were considered higher-confidence potential regulators of these VTGs in SLE blood tissue.

### Kinase activity prediction

Prediction of upstream kinases was performed with iGPS 1.0 software based on short linear motifs at phosphorylation sites [23]. GSEA utilizing kinase-substrate sets (.gmt) and phosphorylation ratios (.gct) inferred kinase activity, with results visualized as a bar plot using R ggplot2.

### Kinase-substrate relationship

NetPhos-3.1 (https://services.healthtech.dtu.dk/services/NetPhos-3.1/) integrated with phosphoproteomic data predicted upstream kinases for differential phosphosites [24, 25]. Consistency between a phosphosite’s change in SLE vs. HC and its kinase’s predicted activity supported the kinase’s role as an upstream regulator.

### Statistical analysis

A false discovery rate (FDR) of 1% was applied for the identification of proteins and phosphorylated peptides. Group differences in the proteomic and phosphoproteomic datasets were assessed using two-tailed Student’s t tests, with a p-value of less than 0.05 considered statistically significant. Data are presented as the mean ± SEM.

## Results

### 4D label-free quantitative analysis reveals abnormal activation of vesicle transport pathways in PBMCs of SLE patients

**Fig. 1:**
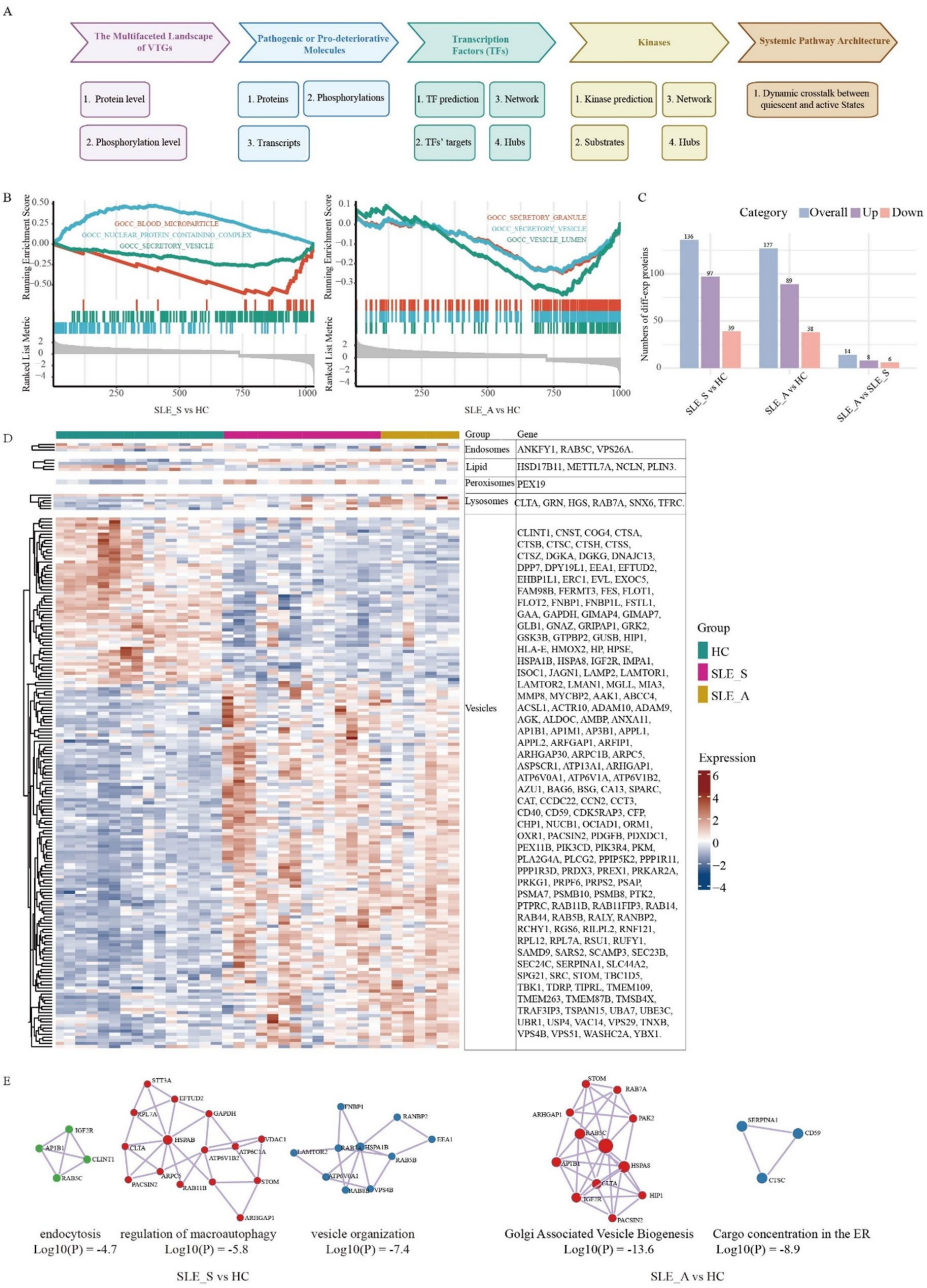
Proteogenomic Characterization of VTGs in PBMCs from Patients with SLE. **A**. A schematic illustrating the overview of this study. **B**. GSEA based on the GOCC database, using all differentially expressed proteins: SLE_S vs. HC (left), SLE_A vs. HC (right) (|NES| > 1.5, *p* < 0.05). **C**. Differential expression patterns of VTGs between disease subgroups. The bar graph displays the number of significantly altered VTGs across three comparisons: SLE_S vs. HC, SLE_A vs. HC, and SLE_A vs. SLE_S. Differential expression is defined as a fold change > 1.5 with a p-value < 0.05. **D**. Categorized expression differences of differentially expressed VTGs are displayed in a heatmap, comparing SLE and HC groups. **E**. PPI network and functional enrichment analysis via Metascape, illustrating the interactions and functional characteristics of differentially expressed VTGs between SLE_S vs. HC (left) and SLE_A vs. HC (right)

To investigate alterations in VTGs in SLE, high-throughput omics analyses were performed on PBMCs from HC and SLE patients. Using 4D label-free quantification (4D-LFQ) technology, proteins were digested with trypsin, enriched by immobilized metal affinity chromatography, and analyzed via liquid chromatography-tandem mass spectrometry. This workflow identified 2,602 proteins and 8,329 phosphorylation sites across 2,797 of these proteins [3] (Supplementary Tables S3 and S4). The transcriptomic data from healthy individuals (*n* = 188) and SLE patients (*n* = 1140) in GEO were integrated to characterize vesicle transport molecular changes across multiple dimensions. Novel phosphorylation modifications, key proteins, and regulatory factors were identified, and pathways associated with VTGs alterations in SLE were delineated. (Fig. 1A).

Based on Gene Set Enrichment Analysis (GSEA) analysis, differentially expressed genes in both SLE_A and SLE_S patients showed significant enrichment in vesicle-related terms from the Gene Ontology Cellular Component (GOCC) database compared to HC (|NES| > 1.5, *p* < 0.05). Specifically, cellular components including blood microparticles, nuclear protein-containing complexes, and secretory vesicles were significantly enriched in SLE_S patients, while secretory granules, secretory vesicles, and vesicle lumen components demonstrated specific enrichment in SLE_A patients (Fig. 1B). These findings collectively suggest widespread functional disturbances in vesicle transport-related cellular structures in PBMCs from SLE patients.

To further elucidate the molecular alterations of VTGs in SLE, we referenced the VTGs gene sets and subclassifications for subsequent analysis. Given that proteins directly perform functions within cells, we first employed proteomic analysis to assess the changes in VTGs in PBMCs from SLE patients. The results revealed that, at the protein level, 97 VTGs were upregulated and 39 were downregulated in the SLE_S vs. HC group; 89 VTGs were upregulated and 38 downregulated in the SLE_A vs. HC group (Fig. 1C).

The heatmap clearly illustrates the differences in abundance of these VTGs, effectively distinguishing between SLE patients and HC (Fig. 1D). In the comparative analysis of SLE vs. HC, we further categorized the expression differences of vesicle transport proteins. The results indicated that 92.47% of the proteins clustered within the vesicle category, followed by lysosomes (3.23%), lipids (2.15%), and endosomes (1.61%), with peroxisomes representing the smallest proportion at just 0.54%. Notable trends of significant upregulation or downregulation of specific proteins emerged among the SLE_A, SLE_S, and HC groups, forming distinct clustering distributions. To further explore the role of VTGs in the pathogenesis and progression of SLE and to elucidate the interactions among these differentially expressed VTGs for identifying potential diagnostic biomarkers with widespread impact in SLE, we.

conducted PPI analysis using the Metascape (Fig. 1E). Notably, protein interaction patterns were highly similar between the active and stable states of SLE. Enrichment analysis revealed that, compared with the healthy control group, the biological processes significantly enriched in SLE_S patients included endocytosis, regulation of macroautophagy, and vesicle organization; while in SLE_A patients, the significantly enriched biological processes primarily involved Golgi-associated vesicle biogenesis and cargo concentration in the endoplasmic reticulum. These findings suggest a distinct expression pattern of vesicle transport genes at the protein level in SLE patients, indicating potential functional disruptions in key biological processes such as organelle transport and autophagy.

### A phosphoproteomics-based comprehensive analysis of the vesicle transport system in SLE immune cells identifies ITSN2 S889 as a potential hub phosphosite

Phosphorylation modifications play crucial roles in regulating protein functions, signaling pathways, and cellular processes, and their dysregulation is closely associated with VTGs functional alterations [26].

**Fig. 2:**
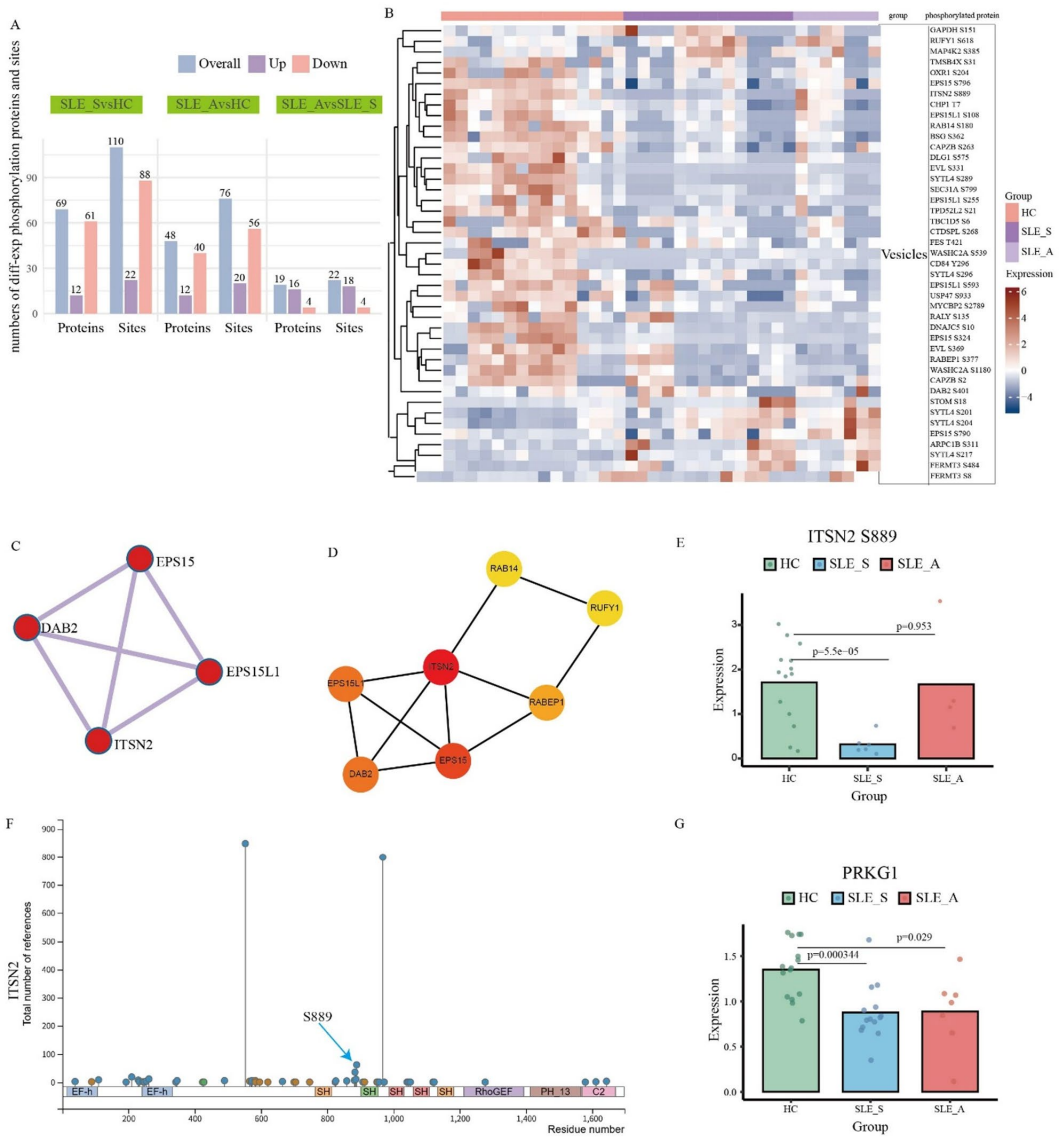
Phosphoproteomic Characterization of VTGs in PBMCs from Patients with SLE. **A**. Quantitative analysis of phosphorylation modifications of VTGs. The bar graph displays the number of significantly altered phosphorylated proteins across three comparisons: SLE_S vs. HC, SLE_A vs. HC, and SLE_A vs. SLE_S. Differential expression is defined as a fold change > 1.5 with a p-value < 0.05. **B**. A heatmap illustrates the expression of phosphorylation sites of differentially expressed VTGs in PBMCs from SLE_S, SLE_A, and HC groups. **C**. Functional enrichment analysis reveals the roles of differentially expressed phosphorylated VTGs in SLE compared to HC. **D**. A network depicting disease-related phosphoprotein interactions of hub VTGs. **E**. Bar graph showing the phosphorylation level of ITSN2 at Ser889 in PBMCs from SLE_S, SLE_A, and HC groups. Data are presented as mean ± SEM, and p-values for group comparisons are indicated. **F**. Mapping of a predicted phosphorylation site (S889) onto the domain structure of ITSN2. **G**. Expression level of the upstream predicted kinase PRKG1 in PBMCs from SLE_S, SLE_A, and HC groups, as determined by proteomic analysis. Data are presented as mean ± SEM, and p-values for group comparisons are indicated

To investigate their potential impact on protein activity, we conducted a phosphoproteomic analysis of VTGs in PBMCs from SLE patients. The results indicated 22 upregulated and 88 downregulated phosphorylation sites in SLE_S vs. HC, and 20 upregulated and 56 downregulated in SLE_A vs. HC (Fig. 2A). We utilized a heatmap to illustrate these differential phosphorylation sites and analyzed their expression across the three groups: SLE_A, SLE_S, and HC (Fig. 2B). The results demonstrated significant differences among the group samples, while within-group samples exhibited considerable consistency.

To further elucidate the biological processes primarily associated with the above-mentioned proteins exhibiting phosphorylation alterations, we performed enrichment analysis using the Metascape database. This analysis identified four densely connected protein groups in the network, consisting of EPS15, EPS15L1, DAB2 and ITSN2 (Fig. 2C). Additionally, we analyzed the PPI network comprised of the differentially expressed VTGs using the CytoHubba plugin in Cytoscape. This analysis revealed that ITSN2, EPS15, EPS15L1, DAB2, RABEP1, RAB14, and RUFY1 were identified as hub genes (Fig. 2D). Intersection analysis yielded a core protein list comprising EPS15, EPS15L1, DAB2, and ITSN2. These proteins exhibit both strong connectivity and high hub scores in the protein-protein interaction network, underscoring their potential relevance to SLE pathogenesis. These findings suggest their potential involvement as key regulatory proteins in the pathological mechanisms of SLE.

The pathogenic roles of several previously mentioned proteins in SLE have been well characterized. For instance, DAB2 is known to mediate the TGF-β signaling pathway and contributes to renal involvement in SLE patients [27]. Both EPS15 [28] and EPS15L1 [29] have also been reported to be associated with SLE, which corroborates the reliability and biological relevance of our screening strategy. Among these four proteins, ITSN2 demonstrated the highest hub score and has attracted significant research interest.

A significant decrease in the phosphorylation of ITSN2 at Ser889 was observed in SLE_S patients compared with HC (Fig. 2E). Interrogation of PhosphoSitePlus [30] for all phosphosites of ITSN2 revealed that S889 is one of the most frequently modified residues (Fig. 2F). The NetPhos-3.1 prediction suggests that PRKG1 can phosphorylate ITSN2 at the Ser889 residue [25]. To investigate whether PRKG1 mediates the phosphorylation of ITSN2 at Ser889 in SLE, we assessed the expression and phosphorylation status of PRKG1. We found that the protein expression of PRKG1 was significantly upregulated in PBMCs from patients with SLE (Fig. 2G), suggesting its potential role as a candidate upstream kinase associated with ITSN2 Ser889 phosphorylation in PBMCs from SLE patients.

### Characterization of early-altered and progression-altered molecules in SLE

**Fig. 3:**
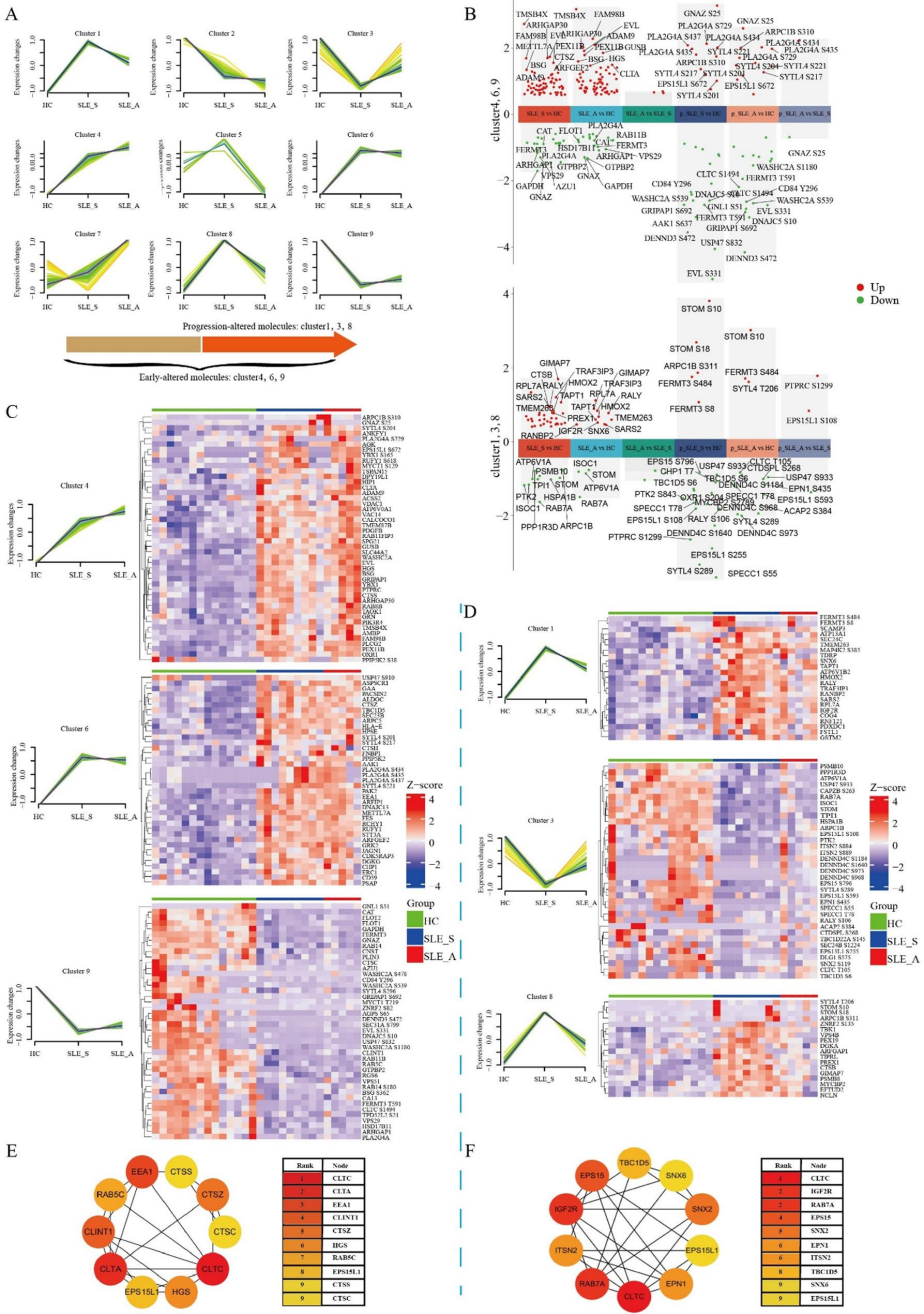
Multi-Omics Integrated Analysis Reveals the Dynamic Regulatory Network of VTGs During SLE Progression. **A**. The Mfuzz time-series clustering plot depicts the expression patterns of VTGs from the proteomics and phosphoproteomics data of SLE_S, SLE_A, and HC groups. **B**. A grouped volcano plot of differential expression features displays the expression levels of VTGs at the protein and phosphorylation levels across different patterns. The x-axis represents the clusters to which the genes belong, while the y-axis indicates the average logFC in gene expression. Red points represent up-regulated genes, and green points represent down-regulated genes. **C-D**. Line graphs and heatmaps show the DEGs across various patterns. The line graph depicts the standardized average expression changes (Z-score) of VTGs across HC, S stage, and A stage, with a dashed line marking the trajectory of disease progression. The heatmap reflects the expression levels of VTGs in HC, S, and A group samples. E-F. PPI networks of differentially expressed genes (DEGs) across various patterns (**E**) and the selection of core driving factors (**F**). The intensity of node color in the PPI network indicates ranking (color intensity: red to yellow signifies scores from high to low). A table lists the top-ranked genes based on their importance within the network

To further elucidate the dynamic changes in VTG expression profiles across different stages of SLE, we performed Mfuzz clustering analysis on VTG-related proteomics and phosphoproteomics data. This analysis categorized all differentially expressed VTGs and their corresponding phosphoproteins into nine distinct expression patterns (Clusters 1–9). The first group comprises clusters 4, 6, and 9. While these clusters show significant gene expression differences between the SLE_S phase and HC, their expression remains stable with no significant differences between the SLE_S and SLE_A phases. These findings suggest these molecules may primarily contribute to disease initiation rather than progression processes. Accordingly, we refer to this group as early-altered molecules. In contrast, the second group includes clusters 1, 3 and 8, where gene expression significantly differs both between SLE_S and HC, and between the S and A phases of SLE. These patterns indicate that the expression of these molecules continues to change as the disease advances. We therefore designate this group as progression-altered molecules (Fig. 3A). Clusters 2, 5 and 7 were excluded from the aforementioned classification due to their scattered expression patterns and undetectable population-wide expression trends. These results indicate that the changes in gene expression during SLE progression exhibit more than a single pattern. Subsequently, we conducted analyses of the VTGs and phosphorylated sites within each group, utilizing volcano plots to illustrate the differences in proteins and phosphorylated protein sites associated with disease progression across various patterns (Fig. 3B). Among the early-altered molecules identified in clusters 4, 6, and 9, downregulated CTSC and RAB family members (RAB5C, RAB11B, RAB14) are functionally linked to neutrophil activity and B/T cell activation/antigen presentation [31, 32]. Meanwhile, the upregulation of HLA-E, PTPRC, and PLA2G4A suggests impaired immune tolerance and renal injury in SLE [33–35]. Additionally, alterations in CLTC S1494 and PLA2G4A S437 may influence SLE progression by modulating inflammatory signaling and antibody production [36, 37]. Based on the expression patterns of proteins and phosphoproteins in clusters 1, 3 and 8 across different SLE stages, we have confirmed their critical roles in disease progression. Notably, PSMB8 and TBK1 are closely associated with antigen processing, interferon regulatory factor activation, and leukocyte adhesion/migration regulation, respectively [38, 39].

The heatmap provides a detailed overview of the expression levels of proteins and phosphorylated protein sites in different samples within the SLE and HC groups, and the observed trends align with the previously established patterns (Fig. 3C and D). Under varying patterns, we conducted protein interaction network analyses using Cytoscape, employing the cytoHubba plugin to depict interactions and identify the top genes associated with the aforementioned changes. Ultimately, we identified two sets of high-scoring genes, with CLTC, CLTA and EEA1 having the highest scores among the early-altered molecules, and CLTC, IGF2R and RAB7A standing out among the progression-altered molecules (Fig. 3E and F). Studies have demonstrated that RAB7A, by regulating the endolysosomal pathway and the maturation of autophagic/endocytic processes, can be inhibited by small molecules to effectively disrupt B cell class switching and plasma cell survival, thereby intervening in the progression of SLE [40]。Despite being categorized into mutually exclusive groups, the protein CLTC (assigned as a Pathogenic Molecule) and its phosphorylated site CLTC T105 (assigned as a Pro-Deteriorative Molecule) each independently achieved the top ranking in their respective groups, indicating their potential role as regulatory candidates in facilitating SLE development through different stages. The discovery of these proteins still provides new directions and potential therapeutic targets for future SLE research.

### Multilevel analysis identifies STAT1 and RELA as key upstream TFs regulating vesicle transport in PBMCs of SLE patients

**Fig. 4:**
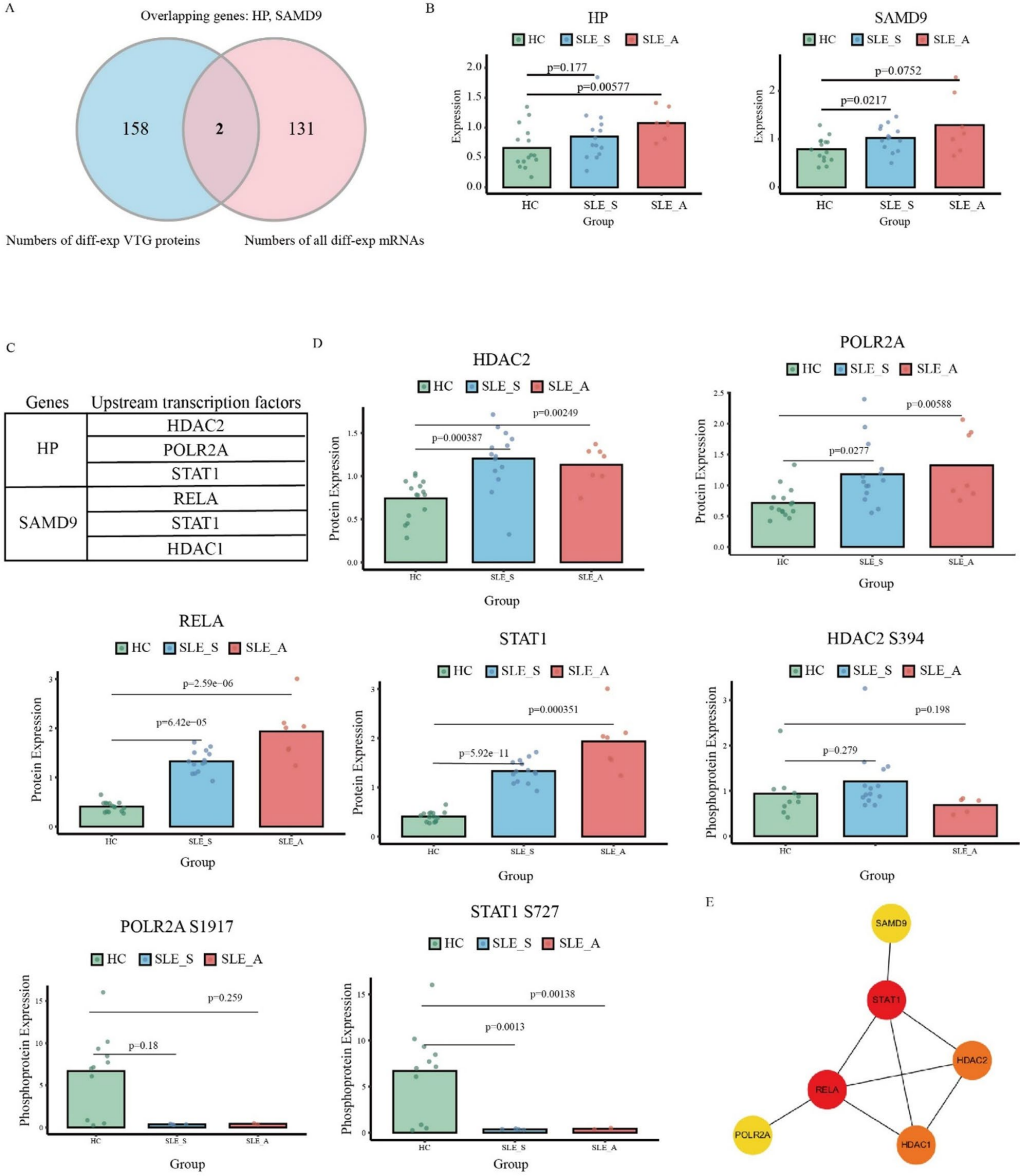
Multi-platform analyses predict key upstream TFs for differentially expressed VTGs in PBMCs of SLE patients. **A**. A Venn diagram shows the overlap in PBMCs between 158 differentially expressed VTGs and 131 differentially expressed mRNAs from SLE patients versus HC. **B**. Protein levels of HP and SAMD9 in PBMCs from SLE_S, SLE_A, and HC groups. Data are presented as mean ± SEM, and p-values for group comparisons are indicated. **C**. The two VTGs, HP and SAMD9, upregulated at both mRNA and protein levels, and their predicted TFs. **D**. Expression levels of TFs HDAC2, POLR2A, RELA, and STAT1 in proteomic and phosphoproteomic data. Data are presented as mean ± SEM, and p-values for group comparisons are indicated. E. PPI network of overlap genes and TFs

In our multi-omics study, proteomics and phosphoproteomics provided insights into protein expression and modifications, enhancing our understanding of critical proteins and their functions in immune responses. However, changes in protein levels alone cannot fully elucidate the molecular mechanisms of disease, particularly at the level of gene expression regulation. To further investigate changes in transcriptional levels, we utilized the RNA-Seq dataset of PBMCs from SLE patients downloaded from GEO to compile a list of genes with significantly differential expression at the mRNA level (Fold change > 1.5, *p* < 0.05). Subsequently, we intersected the differentially expressed mRNAs (*n* = 131) with differentially expressed vesicle transport proteins identified by proteomics (*n* = 158) and found two genes, HP and SAMD9, consistently altered at both transcriptomic and proteomic levels (Fig. 4A). Compared with the HC group, both genes exhibited significant upregulation in either SLE_S or SLE_A groups (Fig. 4B), strongly suggesting their association with SLE pathogenesis and disease activity.

As is well known, TFs exert their effects by regulating the expression of target genes. To ascertain which.

TFs govern the upregulation of the two identified genes, we analyzed potential TFs for these proteins using the hTFtarget database. The results indicate that HP is a target gene of HDAC2, POLR2A, and STAT1, while SAMD9 is targeted by RELA, and STAT1 (Fig. 4C). Subsequently, we further examined the expression levels of these five potential TFs. Our findings revealed that the protein levels of these TFs are significantly upregulated in the PBMCs of SLE patients. Additionally, notable changes in the phosphorylation levels of HDAC2 (S394), POLR2A (S1917), and STAT1 (S727) were observed, while HDAC1 (Q13547) was excluded from presentation due to its undetectable expression in the HC group (Fig. 4D). This further suggests that the expression of the target proteins HP and SAMD9 may be regulated by the expression and phosphorylation status of these TFs.

Additionally, we utilized cytoHubba to identify the core genes within the network of the identified target proteins and TFs. This analysis revealed key hub genes, such as STAT1, RELA, HDAC1, and HDAC2 (Fig. 4E). The critical roles of STAT1 [41] and RELA [42] in immune response and inflammatory regulation in SLE have been extensively reported; however, the subtype-specific mechanisms of HDACs remain unclear and require more refined epigenetic studies. These findings suggest novel disease regulatory pathways and provide a scientific basis for exploring the complex pathogenesis of SLE and developing new therapeutic targets.

### Molecular mechanisms of key kinases and signaling pathways in vesicle transport dysregulation in SLE immune cells

**Fig. 5:**
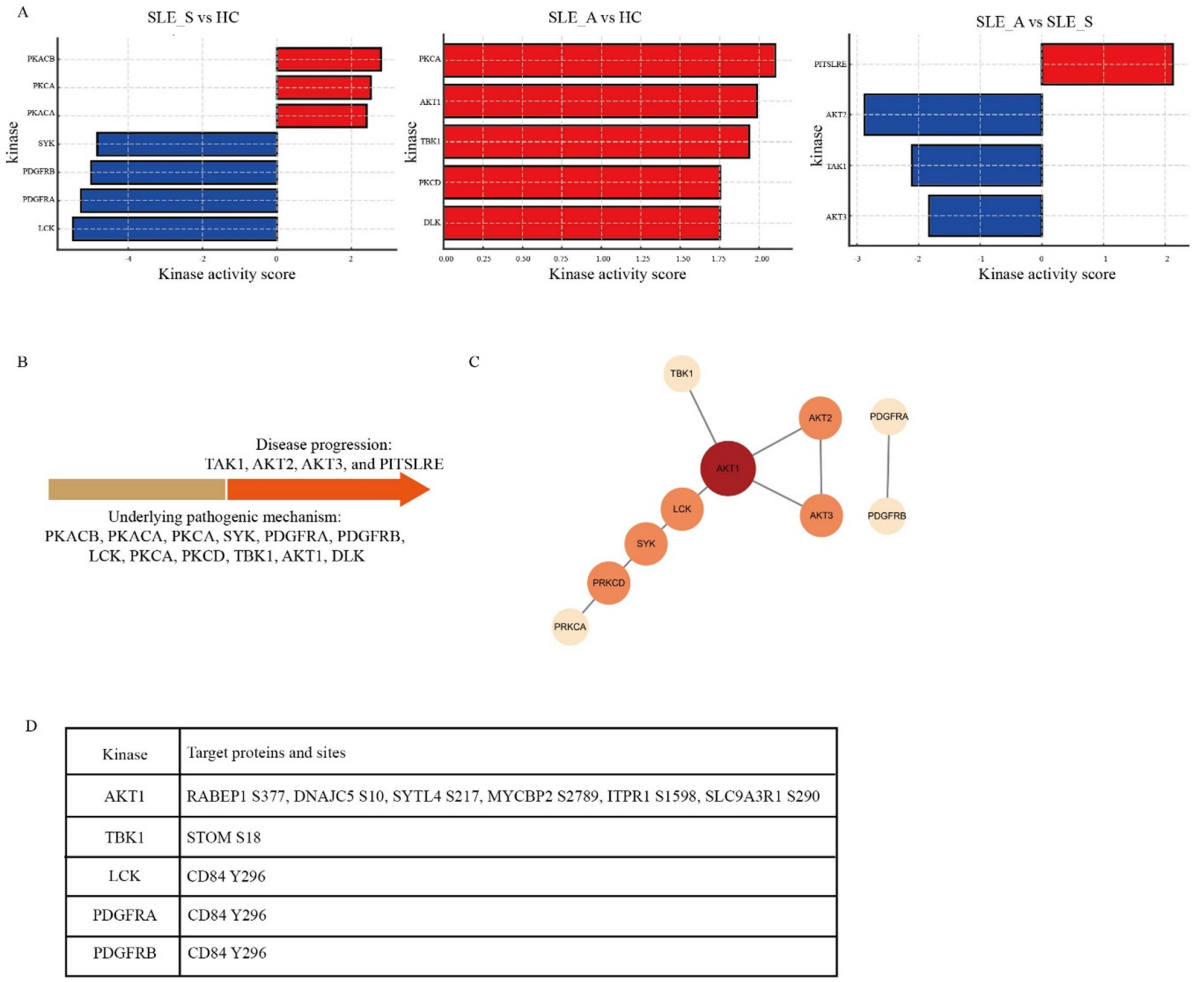
The kinase activity regulatory network suggests putative molecular mechanisms underlying VTGs dysfunction in SLE. **A**. The bar graph displays the activity differences of vesicle-associated kinases across groups: SLE_S vs. HC, SLE_A vs. HC, and SLE_A vs. SLE_S. **B**. Based on differential expression patterns, the kinases were categorized as either disease progression kinases or underlying pathogenic mechanism kinases. **C**. The core kinase interaction network generated by Cytoscape. **D**. Key kinases and their principal phosphosubstrates

Site-specific phosphorylation of proteins is catalyzed by protein kinases (PKs), and these phosphorylation events play a fundamental role in regulating cellular functions, resulting in either the activation or inhibition of signaling pathways. In this study, we predicted the upstream kinases of the phosphorylation sites using sequence similarity principles. Additionally, since the phosphorylation levels may reflect the activity of the kinases, we further assessed kinase activity using kinase-substrate enrichment analysis (KSEA). In the SLE_S vs. HC comparison, PKA family kinases (PKACB, PKACA), PKCA, SYK, type III receptor tyrosine kinases (PDGFRA, PDGFRB), and LCK were identified as significantly altered, indicating early perturbations in cAMP signaling, immune cell activation, and proliferative pathways. In contrast, the SLE_A vs. HC group exhibited increased activity of PKC family kinases (PKCA, PKCD), TBK1, AKT1, and DLK, suggesting a shift toward innate immunity, cell survival, and stress response pathways during the active phase. In the SLE_A vs. SLE_S comparison, TAK1, AKT2, AKT3, and PITSLRE showed differential expression, implicating their roles in disease progression. (Fig. 5A).

Based on the expression dynamics of kinases across groups, we functionally categorized them. TAK1, AKT2, AKT3, and PITSLRE were associated with disease progression, while PKACB, PKACA, PKCA, SYK, PDGFRA, PDGFRB, LCK, PKCA, PKCD, TBK1, AKT1, and DLK were implicated in the underlying pathogenic mechanism (Fig. 5B). Furthermore, cytoHubba analysis identified the core kinases from the aforementioned set as members of the AGC kinase family, namely AKT1, PRKACA, PRKACB, and PRKCA, all of which play important roles in signal transduction (Fig. 5C).

Functional annotation of kinase substrates highlighted key substrates involved in membrane-associated cellular and immune processes. These included RABEP1 S377 (vesicular trafficking and receptor recycling), DNAJC5 S10 (neurotransmitter release and membrane fusion regulation), SYTL4 S217 (vesicle secretion), MYCBP2 S2789 (neuronal development and ubiquitination), ITPR1 S1598 (calcium signaling release), SLC9A3R1 S290 (scaffold function and membrane stability), STOM S18 (ion channel regulation and membrane organization), and the immune-related CD84 Y296 (immune receptor signaling and lymphocyte activation). Together, these results indicate that the kinase network exerts broad and multi-layered regulatory control over vesicular transport, immune responses, and intracellular signaling (Fig. 5D). In summary, this study delineates a dynamic kinase network centered around AKT1 in SLE. This network orchestrates key cellular processes, from vesicular trafficking to immune receptor signaling, thereby presenting a potential therapeutic strategy for targeted kinase inhibition in SLE.

## Discussion

**Fig. 6:**
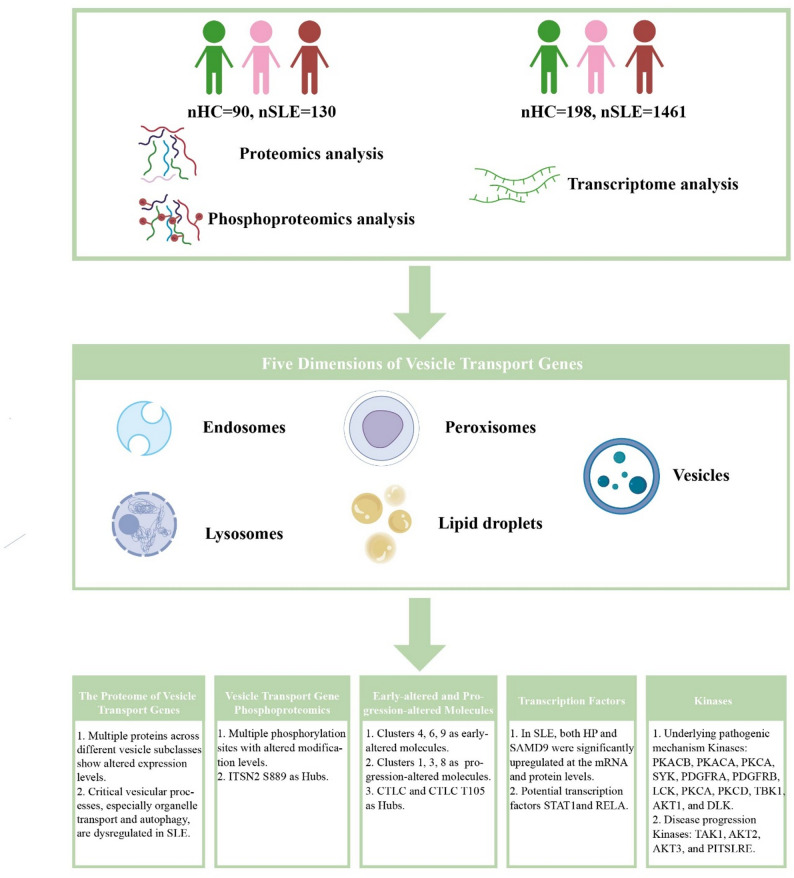
Graphical Abstract. Multi-omics reveals the multidimensional landscape of VTGs in PBMCs from SLE_S, SLE_A, and HC groups

In this study, we performed an integrated analysis of differentially expressed proteomic and phosphoproteomic data related to VTGs in PBMCs from SLE patients (*n* = 130) and HC (*n* = 90), combined with a public GEO dataset (SLE *n* = 1461, HC *n* = 198). After systematically categorizing vesicles into Endosomes, Lysosomes, Peroxisomes, Lipid droplets, and Vesicles, both proteomic and phosphoproteomic analyses revealed significant dysregulation of key vesicular processes, particularly organelle transport and autophagy, in SLE. Phosphoproteomic profiling further identified novel SLE-associated phosphorylation sites, such as S889 in ITSN2. Mfuzz clustering analysis distinguished early-altered molecules from progression-altered molecules. Integrated transcriptomic data suggested that TFs such as STAT1 and RELA may act as upstream regulatory factors modulating the expression of key molecules including HP and SAMD9, and highlighted the potential roles of key kinases including AKT1, PRKACA, PRKACB, and PRKCA. This study systematically elucidates the pathological mechanisms driven by disrupted vesicular transport in SLE, providing potential biomarkers and therapeutic targets for precise treatment (Fig. 6).

Phosphorylation modifications of vesicle transport genes in PBMCs from SLE patients exhibit significant heterogeneity. Further analysis revealed that ITSN2 S889 may function as a potential regulatory node in SLE pathological progression, with its phosphorylation levels showing a consistent association with PRKG1 expression patterns. Previous studies have reported that ITSN2, as a key molecule mediating clathrin-dependent endocytosis, is not only associated with genetic risk for Sjögren’s syndrome and abnormal B-cell methylation [43, 44], but also influences the threshold of autoimmune responses by regulating B/T cell interaction networks [45, 46]. These findings suggest that abnormal phosphorylation of ITSN2 may exacerbate immune dysregulation in SLE by reshaping vesicle-dependent signaling networks, highlighting the critical role of vesicle transport dysfunction in SLE pathogenesis. In summary, our research not only highlights a potential role of ITSN2 S889 phosphorylation in SLE immune dysregulation but also suggests this site may serve as a novel therapeutic target for SLE by modulating vesicular transport pathways.

Through integrated proteomic and phosphoproteomic analyses, we systematically identified multiple core vesicular transport genes in SLE, including CLTC, CLTA, EEA1, IGF2R, and RAB7A. Among these, the CLTC protein and its T105 site rank highly within the interaction network. Studies indicate that CLTC dysfunction disrupts immune cell signaling and antigen presentation efficiency, thereby promoting the activation of autoreactive T/B cells and accelerating SLE progression [37, 47]. Therefore, we hypothesize that CLTC-mediated vesicle transport disruption may serve as a pivotal link connecting innate and adaptive immune dysregulation, offering a novel perspective on understanding the mechanisms underlying SLE’s multi-organ involvement.

By integrating transcriptomic, proteomic, and phosphoproteomic data, we identified significant upregulation of HP and SAMD9 in SLE patients. As hemoglobin-binding proteins, the HP2 allele and HP2-2 genotype are significantly enriched in patients with SLE, rheumatoid arthritis, and inflammatory bowel disease [48, 49]. SAMD9 participates in autoimmune regulation by balancing T cell activation and anti-inflammatory signaling [50, 51]. These findings suggest that HP and SAMD9 may jointly contribute to immune cell dysfunction in SLE patients through distinct immunoregulatory pathways. This study suggests that HP and SAMD9 may serve as potential diagnostic biomarkers for SLE. It also reveals that vesicular transport abnormalities may disrupt common immunoregulatory mechanisms across different autoimmune diseases by affecting these key molecules.

This study further elucidates a critical upstream kinase network closely associated with vesicular transport in SLE. These kinases participate in autoimmune pathogenesis by regulating immune cell proliferation, apoptosis, migration, and inflammatory signaling cascades. Notably, similar kinase activity patterns have been observed in multiple sclerosis [52, 53], suggesting that kinase-dependent phosphorylation networks may represent a common regulatory mechanism across autoimmune diseases. In SLE, the AGC kinase network centered on AKT1, PKA, and PKC achieves multi-level precise regulation of the vesicle transport-immune activation axis by phosphorylating key vesicle transport proteins such as RABEP1 and DNAJC5, as well as immune signaling molecules like CD84. Dysregulation of this network constitutes an important driver of immune dysfunction in SLE, providing direct theoretical justification for developing targeted therapies against this kinase network.

Conceptually, it is important to distinguish the advancement of this study from our previous global phosphoproteomic characterization. While the foundational dataset shares similarities in cohort origin, the scientific objectives and analytical depth differ fundamentally. Our previous atlas served as a broad-scale resource to identify systemic dysregulation; in contrast, this study moves beyond mere cataloging of phosphorylation events to provide a mechanism-oriented reconstruction of the vesicle transport-immune activation axis. By integrating multi-layered omics data, we transitioned from a descriptive “what” (the landscape of alterations) to a mechanistic “how” (how upstream kinases coordinate vesicle-specific regulatory hubs). This shift establishes a dynamic, high-resolution, and biologically interpretable landscape of VTG regulation that was previously obscured in broad-scale profiling.

By shifting from a static global overview to a trajectory-based analytical framework, we provide a dynamic, high-resolution, and biologically interpretable landscape of VTG regulation that was previously obscured in broad-scale profiling. First, regarding the experimental design, we acknowledge the limitations inherent to the sample pooling strategy. While this approach was necessitated by the stringent protein input requirements of deep phosphoproteomics (~ 1 mg) and the clinical challenge of obtaining sufficient PBMCs from lymphopenic SLE patients, it inevitably masks inter-individual heterogeneity. Consequently, this design restricted our ability to detect rare patient-specific regulatory events and precluded direct correlation analyses between specific phosphosite alterations and individual clinical indices, such as SLEDAI-2 K scores and serological markers. To mitigate these concerns, we integrated our phosphoproteomic data with large-scale external transcriptomic datasets at the individual level, observing a high degree of pathway concordance that supports the biological validity of our pooled findings. Future studies utilizing single-sample phosphoproteomics or targeted validation (e.g., phospho-specific Western blotting) will be essential to fully characterize SLE heterogeneity and establish direct links between kinase signaling and disease activity.

## Conclusion

In conclusion, employing 4D label-free quantitative analysis, we systematically profiled VTGs in PBMCs from SLE patients at both the proteomic, phosphoproteomic and transcriptomic levels. This approach facilitated the identification of abnormal activation within multidimensional VTG-related pathways and uncovered novel regulatory targets. Our results not only validate established pathogenic pathways but also delineate novel molecular mechanisms, thereby offering valuable insights for the future development of precise diagnostics and targeted therapies for SLE.

## Supplementary Information

Supplementary Material 1

Supplementary Material 2

Supplementary Material 3

Supplementary Material 4

## Data Availability

The proteomic and phosphoproteomic data used in this study were generated in our laboratory[3]. The proteomic and phosphoproteomic datasets used in this study were produced by our laboratory. The datasets generated during and/or analysed during the current study are available in the ProteomeXchange repository with the accession number PXD025559. All differentially expressed phosphosites and proteins are listed in Supplementary Table S1-2.

## Abbreviations

SLE: Systemic lupus erythematosus
VTGs: Vesicle transport genes
PPI: Protein-protein Interaction
PBMCs: Peripheral blood mononuclear cells
HC: Healthy controls
4D-LFQ: 4D label-free quantification
GSEA: Gene Set Enrichment Analysis
GOCC: Gene Ontology Cellular Component
TFs: Transcription factors
RA: Rheumatoid arthritis

## References

[1] Tsokos ADDINENREFLIST. Autoimmunity and organ damage in systemic lupus erythematosus. Nat Immunol. 2020;21(6):605–14. 32367037 10.1038/s41590-020-0677-6 PMC8135909

[2] Stojan G, Petri M. Epidemiology of systemic lupus erythematosus: an update. Curr Opin Rheumatol. 2018;30(2):144–50. 29251660 10.1097/BOR.0000000000000480 PMC6026543

[3] Meng S, Li T, Wang T, Li D, Chen J, Li H, et al. Global phosphoproteomics unveils kinase-regulated networks in systemic lupus erythematosus. Mol Cell Proteomics. 2022;21(12):100434. 36309313 10.1016/j.mcpro.2022.100434 PMC9712766

[4] Patiño-Martinez E, Kaplan MJ. Immunometabolism in systemic lupus erythematosus. Nat Rev Rheumatol. 2025;21(7):377–95. 40524030 10.1038/s41584-025-01267-0

[5] Richter P, Macovei LA, Mihai IR, Cardoneanu A, Burlui MA, Rezus E. Cytokines in systemic lupus erythematosus-focus on TNF-α and IL-17. Int J Mol Sci. 2023;24(19):14413. 37833861 10.3390/ijms241914413 PMC10572174

[6] Crow MK. Pathogenesis of systemic lupus erythematosus: risks, mechanisms and therapeutic targets. Ann Rheum Dis. 2023;82(8):999. 36792346 10.1136/ard-2022-223741

[7] Su X, Yu H, Lei Q, Chen X, Tong Y, Zhang Z, et al. Systemic lupus erythematosus: pathogenesis and targeted therapy. Mol Biomed. 2024;5(1):54. 39472388 10.1186/s43556-024-00217-8 PMC11522254

[8] Wu WC, Song SJ, Zhang Y, Li X. Role of extracellular vesicles in autoimmune pathogenesis. Front Immunol. 2020;11:579043. 33072123 10.3389/fimmu.2020.579043 PMC7538611

[9] Liu Y-J, Wang C. A review of the regulatory mechanisms of extracellular vesicles-mediated intercellular communication. Cell Commun Signal. 2023;21(1):77. 37055761 10.1186/s12964-023-01103-6 PMC10100201

[10] Lee JY, Park JK, Lee EY, Lee EB, Song YW. Circulating exosomes from patients with systemic lupus erythematosus induce a proinflammatory immune response. Arthritis Res Ther. 2016;18:1–8. 27852323 10.1186/s13075-016-1159-y PMC5112700

[11] Ratajczak J, Wysoczynski M, Hayek F, Janowska-Wieczorek A, Ratajczak MZ. Membrane-derived microvesicles: important and underappreciated mediators of cell-to-cell communication. Leukemia. 2006;20(9):1487–95. 16791265 10.1038/sj.leu.2404296

[12] Nielsen CT, Østergaard O, Stener L, Iversen LV, Truedsson L, Gullstrand B, et al. Increased IgG on cell-derived plasma microparticles in systemic lupus erythematosus is associated with autoantibodies and complement activation. Arthritis Rheumatol. 2012;64(4):1227–36. 10.1002/art.34381 22238051

[13] Park JS, Perl A. Endosome traffic modulates pro-inflammatory signal transduction in CD4+ T cells—implications for the pathogenesis of systemic lupus erythematosus. Int J Mol Sci. 2023;24(13):10749. 37445926 10.3390/ijms241310749 PMC10341602

[14] Holcombe RF, Baethge BA, Wolf RE, Betzing KW, Stewart RM, Hall VC, et al. Correlation of serum Interleukin-8 and cell surface lysosome-associated membrane protein with clinical disease activity in systemic lupus erythematosus. Lupus. 1994;3(2):97–102. 7920621 10.1177/096120339400300207

[15] Ge W, Li D, Gao Y, Cao X. The roles of lysosomes in inflammation and autoimmune diseases. Int Rev Immunol. 2015;34(5):415–31. 25075736 10.3109/08830185.2014.936587

[16] Rőszer T, Menéndez-Gutiérrez MP, Lefterova MI, Alameda D, Núñez V, Lazar MA, Fischer T, Ricote M. Autoimmune kidney disease and impaired engulfment of apoptotic cells in mice with macrophage peroxisome proliferator-activated receptor γ or retinoid X receptor α deficiency. J Immunol. 2011;186(1):621–31. 21135166 10.4049/jimmunol.1002230 PMC4038038

[17] Zhong Y, Zhang W, Hong X, Zeng Z, Chen Y, Liao S, et al. Screening biomarkers for systemic lupus erythematosus based on machine learning and exploring their expression correlations with the ratios of various immune cells. Front Immunol. 2022;13:873787. 35757721 10.3389/fimmu.2022.873787 PMC9226453

[18] Zhou Y, Zhou B, Pache L, Chang M, Khodabakhshi AH, Tanaseichuk O, et al. Metascape provides a biologist-oriented resource for the analysis of systems-level datasets. Nat Commun. 2019;10(1):1523. 30944313 10.1038/s41467-019-09234-6 PMC6447622

[19] Szklarczyk D, Gable AL, Lyon D, Junge A, Wyder S, Huerta-Cepas J, Simonovic M, Doncheva NT, Morris JH, Bork P. STRING v11: protein–protein association networks with increased coverage, supporting functional discovery in genome-wide experimental datasets. Nucleic Acids Res. 2019;47(D1):D607–13. 30476243 10.1093/nar/gky1131 PMC6323986

[20] Shannon P, Markiel A, Ozier O, Baliga NS, Wang JT, Ramage D, Amin N, Schwikowski B, Ideker T. Cytoscape: a software environment for integrated models of biomolecular interaction networks. Genome Res. 2003;13(11):2498–504. 14597658 10.1101/gr.1239303 PMC403769

[21] Kumar L. Mfuzz: a software package for soft clustering of microarray data. Bioinformation. 2007;2(1):5–7. 18084642 10.6026/97320630002005 PMC2139991

[22] Zhang Q, Liu W, Zhang H-M, Xie G-Y, Miao Y-R, Xia M, et al. Bioinformatics: hTFtarget: a comprehensive database for regulations of human transcription factors and their targets. Genom Proteom Bioinform. 2020;18(2):120–8. 10.1016/j.gpb.2019.09.006 PMC7647694 32858223

[23] Song C, Ye M, Liu Z, Cheng H, Jiang X, Han G, Songyang Z, Tan Y, Wang H, Ren J. Systematic analysis of protein phosphorylation networks from phosphoproteomic data. Mol Cell Proteom. 2012;11(10):1070–83. 10.1074/mcp.M111.012625 PMC3494146 22798277

[24] Blom N, Gammeltoft S, Brunak S. Sequence and structure-based prediction of eukaryotic protein phosphorylation sites. J Mol Biol. 1999;294(5):1351–62. 10600390 10.1006/jmbi.1999.3310

[25] Blom N, Sicheritz-Pontén T, Gupta R, Gammeltoft S, Brunak S. Prediction of post‐translational glycosylation and phosphorylation of proteins from the amino acid sequence. Proteomics. 2004;4(6):1633–49. 15174133 10.1002/pmic.200300771

[26] Minic Z, Hüttmann N, Poolsup S, Li Y, Susevski V, Zaripov E, et al. Phosphoproteomic analysis of breast cancer-derived small extracellular vesicles reveals disease-specific phosphorylated enzymes. Biomedicines. 2022;10(2):408. 35203617 10.3390/biomedicines10020408 PMC8962341

[27] Zhang J, Chang L, Sun Y, Qin M, Wang X, Guo Y. Disabled-2 overexpression mediates immune suppression in systemic lupus erythematosus by modulating Treg/Th17 cell differentiation. Clin Exp Pharmacol Physiol. 2022;49(5):596–607. 35108421 10.1111/1440-1681.13630

[28] Pozzi B, Amodio S, Lucano C, Sciullo A, Ronzoni S, Castelletti D, et al. The endocytic adaptor Eps15 controls marginal zone B cell numbers. PLoS One. 2012;7(11):e50818. 23226392 10.1371/journal.pone.0050818 PMC3511280

[29] Milesi C, Alberici P, Pozzi B, Oldani A, Beznoussenko GV, Raimondi A, et al. Redundant and nonredundant organismal functions of EPS15 and EPS15L1. Life Sci Alliance. 2019;2(1):e201800273. 10.26508/lsa.201800273. 30692166 10.26508/lsa.201800273 PMC6350104

[30] Hornbeck PV, Zhang B, Murray B, Kornhauser JM, Latham V, Skrzypek E. PhosphoSitePlus, 2014: mutations, PTMs and recalibrations. Nucleic Acids Res. 2015;43(D1):D512–20. 25514926 10.1093/nar/gku1267 PMC4383998

[31] Lv F, Li X, Wang Z, Wang X, Liu J. Identification and validation of Rab GTPases RAB13 as biomarkers for peritoneal metastasis and immune cell infiltration in colorectal cancer patients. Front Immunol. 2024;15:1403008. 39192986 10.3389/fimmu.2024.1403008 PMC11347351

[32] He Y, Huang M, Wang Y, Cai X, Xiao F. Inhibition of CTSC contributes to psoriasis inflammation and keratinocyte hyperproliferation by NF-κB signaling pathway. Int Immunopharmacol. 2025;157:114808. 40339488 10.1016/j.intimp.2025.114808

[33] Jucaud V, Ravindranath MH, Terasaki PI, Morales-Buenrostro LE, Hiepe F, Rose T, et al. Serum antibodies to human leucocyte antigen (HLA)-E, HLA-F and HLA-G in patients with systemic lupus erythematosus (SLE) during disease flares: clinical relevance of HLA-F autoantibodies. Clin Exp Immunol. 2016;183(3):326–40. 26440212 10.1111/cei.12724 PMC4750595

[34] Qian D, Liu L, Zhu T, Wen L, Zhu Z, Yin X, et al. JAK2 and PTPRC mRNA expression in peripheral blood mononuclear cells from patients with systemic lupus erythematosus. Clin Rheumatol. 2020;39(2):443–8. 31760539 10.1007/s10067-019-04778-w

[35] Luo Y, Liu L, Zhang C. Identification and analysis of diverse cell death patterns in diabetic kidney disease using microarray-based transcriptome profiling and single-nucleus RNA sequencing. Comput Biol Med. 2024;169:107780. 38104515 10.1016/j.compbiomed.2023.107780

[36] Qian Y, Yuan Q, Yu H, Ye R, Niu M, Liu F. Multi-omics analysis of the effects of pla2g4a on the prognosis of various cancers and its experimental validation in breast cancer cell lines. Discov Oncol. 2025;16(1):1278. 40624348 10.1007/s12672-025-03118-6 PMC12234970

[37] Xie T, Dong J, Zhou X, Tang D, Li D, Chen J, et al. Proteomics analysis of lysine crotonylation and 2-hydroxyisobutyrylation reveals significant features of systemic lupus erythematosus. Clin Rheumatol. 2022;41(12):3851–8. 35941338 10.1007/s10067-022-06254-4 PMC9652266

[38] Shi Y, Guan S, Liu X, Zhai H, Zhang Y, Liu J, et al. Genetic commonalities between metabolic syndrome and rheumatic diseases through disease interactome modules. J Cell Mol Med. 2025;29(1):e70329. 39789419 10.1111/jcmm.70329 PMC11717667

[39] Pan M, Yin Y, Hu T, Wang X, Jia T, Sun J, et al. UXT attenuates the CGAS-STING1 signaling by targeting STING1 for autophagic degradation. Autophagy. 2023;19(2):440–56. 35543189 10.1080/15548627.2022.2076192 PMC9851252

[40] Lam T, Kulp DV, Wang R, Lou Z, Taylor J, Rivera CE, et al. Small molecule inhibition of Rab7 impairs B cell class switching and plasma cell survival to dampen the autoantibody response in murine lupus. J Immunol. 2016;197(10):3792–805. 27742832 10.4049/jimmunol.1601427 PMC5113143

[41] Yiu G, Rasmussen TK, Tsai BL, Diep VK, Haddon DJ, Tsoi J, et al. High interferon signature leads to increased STAT1/3/5 phosphorylation in PBMCs from SLE patients by single cell mass cytometry. Front Immunol. 2022;13:833636. 35185925 10.3389/fimmu.2022.833636 PMC8851522

[42] Mao H, Zhao X, Sun S-c. NF-κB in inflammation and cancer. Cell Mol Immunol. 2025;22(8):811–39. 40562870 10.1038/s41423-025-01310-w PMC12310982

[43] Lessard CJ, Li H, Adrianto I, Ice JA, Rasmussen A, Grundahl KM, et al. Variants at multiple loci implicated in both innate and adaptive immune responses are associated with Sjögren’s syndrome. Nat Genet. 2013;45(11):1284–92. 24097067 10.1038/ng.2792 PMC3867192

[44] Miceli-Richard C, Wang-Renault S-F, Boudaoud S, Busato F, Lallemand C, Bethune K, et al. Overlap between differentially methylated DNA regions in blood B lymphocytes and genetic at-risk loci in primary Sjögren's syndrome. Ann Rheum Dis. 2016;75(5):933. 26183421 10.1136/annrheumdis-2014-206998 PMC4853580

[45] Burbage M, Keppler SJ. Shaping the humoral immune response: Actin regulators modulate antigen presentation and influence B-T interactions. Mol Immunol. 2018;101:370–6. 30055407 10.1016/j.molimm.2018.07.026

[46] Burbage M, Gasparrini F, Aggarwal S, Gaya M, Arnold J, Nair U, Way M, Bruckbauer A, Batista FD. Tuning of in vivo cognate B-T cell interactions by Intersectin 2 is required for effective anti-viral B cell immunity. eLife. 2018;7:e26556. 29337666 10.7554/eLife.26556 PMC5770159

[47] Celhar T, Hopkins R, Thornhill SI, De Magalhaes R, Hwang S-H, Lee H-Y, et al. RNA sensing by conventional dendritic cells is central to the development of lupus nephritis. Proc Natl Acad Sci U S A. 2015;112(45):E6195–204. 26512111 10.1073/pnas.1507052112 PMC4653170

[48] Vanuytsel T, Vermeire S, Cleynen I. The role of Haptoglobin and its related protein, Zonulin, in inflammatory bowel disease. Tissue Barriers. 2013;1(5):e27321. 24868498 10.4161/tisb.27321 PMC3943850

[49] Van Vlierberghe H, Langlois M, Delanghe J. Haptoglobin polymorphisms and iron homeostasis in health and in disease. Clin Chim Acta. 2004;345(1–2):35–42. 15193975 10.1016/j.cccn.2004.03.016

[50] He P, Wu L-F, Bing P-F, Xia W, Wang L, Xie F-F, Lu X, Lei S-F, Deng F-Y. SAMD9 is a (epi-) genetically regulated anti-inflammatory factor activated in RA patients. Mol Cell Biochem. 2019;456(1):135–44. 30715670 10.1007/s11010-019-03499-7

[51] Peng S, Meng X, Zhang F, Pathak PK, Chaturvedi J, Coronado J, et al. Structure and function of an effector domain in antiviral factors and tumor suppressors SAMD9 and SAMD9L. Proc Natl Acad Sci U S A. 2022;119(4):e2116550119. 35046037 10.1073/pnas.2116550119 PMC8795524

[52] Tsai HF, Lai JJ, Chou AH, Wang TF, Wu CS, Hsu PN. Induction of costimulation of human CD4 T cells by tumor necrosis factor–related apoptosis-inducing ligand: Possible role in T cell activation in systemic lupus erythematosus. Arthritis Rheumatism: Official J Am Coll Rheumatol. 2004;50(2):629–39. 10.1002/art.20038 14872508

[53] Cuenda A, Rousseau S. P38 MAP-kinases pathway regulation, function and role in human diseases. Biochimica et Biophysica Acta (BBA) - Molecular Cell Research. 2007;1773(8):1358–75. 17481747 10.1016/j.bbamcr.2007.03.010

